# Is It Right to Vaccinate or Revaccinate Pregnant Women?

**Published:** 1871-06

**Authors:** 


### Is it Right to Vaccinate or Revaccinate Pregnant Women?

Dr. Robert Barnes, of London (*British Medical Journal*), in an-answer to this question, states that so far is vaccinatton from causing abortion, cases are known in in which the fœtus has gone safely through the vaccine disease *in utero*, so that it has subsequently been proof against vaccination. He believes we may conclude, in the absence of decisive evidence of special danger, that pregnant woman are entitled to equal protection against smallpox with the rest of the community; and that vaccination or revaccination should be practised on pregnant women, in their own interest as well as that of the community of which they form a part.

